# Genomic Instability of I Elements of *Drosophila melanogaster* in Absence of Dysgenic Crosses

**DOI:** 10.1371/journal.pone.0013142

**Published:** 2010-10-04

**Authors:** Roberta Moschetti, Patrizio Dimitri, Ruggiero Caizzi, Nikolaj Junakovic

**Affiliations:** 1 Dipartimento di Genetica e Microbiologia, Università di Bari “Aldo Moro”, Bari, Italy; 2 Dipartimento di Genetica e Biologia Molecolare, Charles Darwin, Roma, Italy; 3 Istituto di Biologia e Patologia Molecolari, CNR, Roma, Italy; Ludwig-Maximilians-Universität München, Germany

## Abstract

Retrotranspostion of I factors in the female germline of *Drosophila melanogaster* is responsible for the so called I-R hybrid dysgenesis, a phenomenon that produces a broad spectrum of genetic abnormalities including reduced fertility, increased frequency of mutations and chromosome loss. Transposition of I factor depends on cellular conditions that are established in the oocytes of the reactive females and transmitted to their daughters. The so-called reactivity is a cellular state that may exhibit variable levels of expression and represents a permissive condition for I transposition at high levels. Defective I elements have been proposed to be the genetic determinants of reactivity and, through their differential expression, to modulate transposition of active copies in somatic and/or germ line cells. Recently, control of transposable element activity in the germ line has been found to depend on pi-RNAs, small repressive RNAs interacting with Piwi-family proteins and derived from larger transposable elements (TE)-derived primary transcripts. In particular, maternally transmitted I-element piRNAs originating from the 42AB region of polytene chromosomes were found to be involved in control of I element mobility. In the present work, we use a combination of cytological and molecular approaches to study the activity of I elements in three sublines of the inducer *y*; *cn bw*; *sp* isogenic strain and in dysgenic and non-dysgenic genetic backgrounds. Overall, the results of FISH and Southern blotting experiments clearly show that I elements are highly unstable in the Montpellier subline in the absence of classical dysgenic conditions. Such instability appears to be correlated to the amount of 5′ and 3′ I element transcripts detected by quantitative and real-time RT-PCR. The results of this study indicate that I elements can be highly active in the absence of a dysgenic crosses. Moreover, in the light of our results caution should be taken to assimilate the genomic annotation data on transposable elements to all *y*; *cn bw sp* sublines.

## Introduction

*I* factors of *Drosophila melanogaster* are LINE-like elements that actively transpose in the germline of the female progeny (called SF) from crosses between females of reactive (R) strains and males of inducer (I) strains [1]; for a recent review see [2]. This phenomenon, called I-R hybrid dysgenesis, gives rise to reduced fertility, X chromosome loss and increased frequency of euchromatic and heterochromatic mutations. Inducer strains contain 10–15 euchromatic dispersed functional copies of the *I* factor, a 5.4 kb DNA sequence; in addition they carry multiple copies of defective *I* elements embedded in heterochromatin. By contrast, reactive strains only contain the defective heterochromatic copies and lack functional elements [1], [3]. In both reactive and inducer strains defective *I* elements form prominent clusters within the heterochromatin of mitotic chromosomes at multiple locations which are stable in unrelated strains [4].

Transposition of *I* factor depends on cellular conditions that are established in the oocytes of the reactive females and transmitted to their daughters. The so-called reactivity is a cellular state that may be expressed at variable levels [5] and represents a permissive condition for *I* transposition at high levels. It has been proposed that defective *I* elements act as the genetic determinants of reactivity and, through their differential expression, can modulate transposition of active copies [6], [7]. Several lines of experimental evidence subsequently confirmed that prediction, showing that defective *I* elements are indeed involved in homology-dependent silencing of *I* factor-related sequences [8], [9], [10], [11], [12], [13], [14]. *I* element-homologous sequences, possibly involved in silencing of I factor, have been mapped by FISH to region h28 of the X- chromosome mitotic heterochromatin [15]. More recently, control of retrotransposon activity in the germ line has been ascribed to small RNAs interacting with Piwi-family proteins (Piwi, Aubergine, and AGO3), called piRNAs [16], [17]. In particular, maternally transmitted I-element piRNAs originated from 42AB region of polytene chromosomes were found to be involved in the control of *I* element mobility [18].

Previous cytogenetic data suggested that *I* elements can move in the absence of dysgenic conditions in natural populations of *Drosophila melanogster* [19]. Instability of *I* elements and other TEs has also been detected in several *Drosophila melanogaster* stocks that were expected to be stable as they had been rendered isogenic by various approaches [20], [21], [22], [23]. By Southern blotting a highly polymorphic pattern of *I* elements was revealed among individuals of a highly inbred line (N. Junakovic, unpublished). Zakharenko et al. [24] extended these observations further to a *y*; *cn bw sp* strain used for the *Drosophila melanogaster* genome sequencing [25]. This strain is suitable for studying novel changes in the genomic distribution of TEs due to isogenization and presence of phenotypic markers which enable us to distinguish transpositional events from drift of preexisting polymorphisms or contamination.

In this work, we have analysed individual flies from different sublines of the isogenic inducer strain *y*; *cn bw sp* by Southern blotting, FISH and RT-PCR to assess the functional status of *I* elements and other TE families. The results indicate that *I* elements can be highly active in the absence of a dysgenic background.

## Results

### Southern blotting analysis of *I* element genomic distribution in isogenic lines

**Figure 1:**
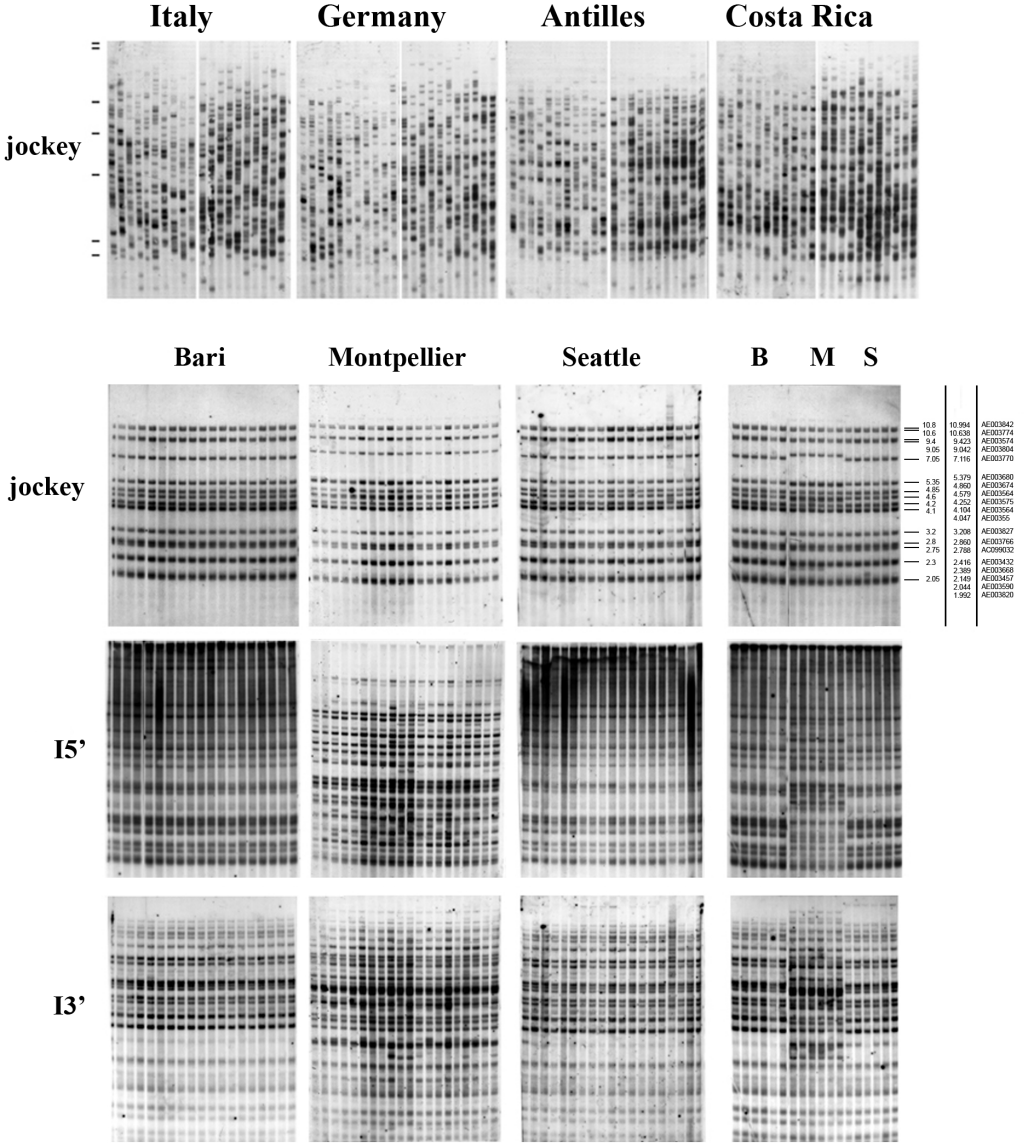
*Jockey* and *I* element distribution in *y*; *cn bw*; *sp* sublines. Individual flies of Bari (B), Montpellier (M) and Seattle (S) sublines of the *y*; *cn bw*; *sp* strain and from four natural populations were analyzed by the Southern blotting technique. The same filters have been sequentially tested with probes homologous to *jockey* and the two ends of *I* transposable elements. The *jockey* patterns are very similar in the 3 sublines and significantly overlap in number and mobility of bands with the virtual pattern retrieved from the sequenced Drosophila genome, as expected for an isogenic strain (see size markers and Table 1). By converse, *jockey* is highly variable among individuals of four geographically different populations recently collected from the wild (see top panels). After stripping the same filters from the *jockey* signal and reprobing them sequentially with the two end of *I* elements, Bari and Seattle sublines patterns appear homogeneous within and between the stocks; on the contrary the Montpellier individuals are heterogeneous between them and the overall stock differs from the previous two patterns. This is best appreciated in the right handside picture labelled B M S, bearing individuals of the 3 stocks on the same filter.

We focused on three different sublines of the *y*; *cn bw sp* strain, called Bari, Montpellier and Seattle, all originating from the Bloomington stock center and independently maintained for some years in the laboratories of Ruggiero Caizzi (Bari), Alain Bucheton (Montpellier) and Barbara Wakimoto (Seattle). Genomic DNA was extracted from individual flies of these sublines and tested by Southern blotting using *jockey* and *I* element probes. As shown in the top of Figure 1, *jockey* showed a highly variable pattern among individuals of four geographically different populations recently collected from the wild, while producing the same pattern of fragments among individual flies from the *y; cn bw sp* sublines, as expected for an isogenic strain (Fig. 1). In particular, the number and mobility of fragments obtained with the *jockey* probes matches their respective virtual counterparts, as retrieved in the sequenced genome (Fig. 1 and Table 1). That observation demonstrates that *jockey* elements remained stable in the three sublines at least since the time of DNA extraction from the *y*; *cn bw sp* strain for the genome sequencing project. Thus, the *jockey* pattern represents a kind of fingerprint that identifies the sublines as identical to the *y*; *cn bw sp* sequenced strain and provides a useful molecular marker, in addition to phenotypic markers, as a control against contamination.

**Table 1:** Comparison of *jockey* HindIII fragments obtained by Southern and *in silico.*

kb Southern	Kb *In silico*	chromosome	start	end
10,8010,61	10,73310,994	2R2R	142587543123723	142597583125343
9,40	9,423	X	21967238	21968858
9,05	9,042	2R	13037805	13039425
7,05	7116	3R	25479232	25480852
5,35	5,379	3R	4533548	4630591
4,85	4,860	3R	2336147	2337767
4,60	4,579	3L	26306697	26308317
4,26	4,300	2R	14249021	14249636
4,24	4,252	2L	4921270	4922890
4,15	4,047	3L	9572121	9573741
nd	4,005	3R	24079459	24081079
3,20	3,208	2R	6910509	6912129
2,84	2,860	3R	24618986	24620606
2,75	2,772	X	1412540	1414160
2,402,312,11	2,389ndnd	2L	20894671	20896291
2,05nd	2,0441,992	2L2R	478628707349	494828708969

Start and end define the genomic coordinates of *the in silico* fragments.

kb =  kilobases; nd =  not detected

In contrast to the *jockey* stability, sequential hybridization of the same filters with both 5′ and 3′ end probes of the *I* element revealed a clear heterogeneity of the pattern of fragments among individual flies in the Montpellier subline, the other two sublines being identical (Fig. 1). This result indicates that the *I* element can be unstable in *y*; *cn bw sp* sublines.

**Figure 2:**
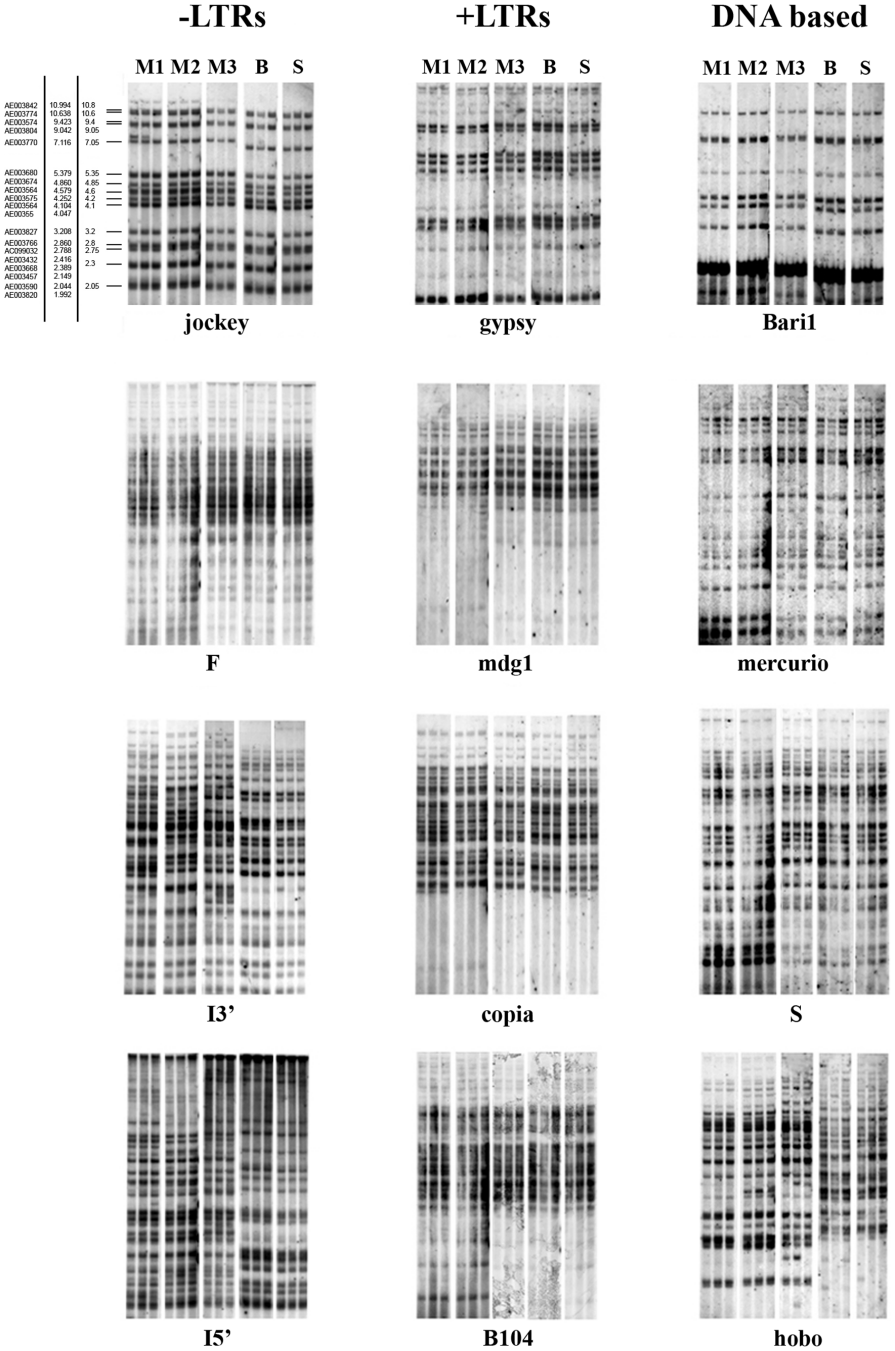
Southern blotting analysis of retroelements and DNA transposons distribution in Montpellier, Bari, and Seattle sublines. In addition to *jockey* and *I* element probes, the distribution of five retroelements and four DNA transposons was studied among individual flies of the 3 sublines. In M1, M2 and M3 DNA from different generations of Montpellier was analysed. The comparison of M1, M2 and M3 shows de novo instability of *jockey*, *I* element and *hobo* generated during the time in Montpellier. -LTR  =  elements without LTRs; +LTR  =  elements with LTRs).

In order to assess whether the genomic instability involves other TE families, genomic DNA extracted from individual flies of the three *y*; *cn bw sp* sublines was further tested by Southern with probes from nine different TEs, together with the *I* (both 5′ and 3′ probes) and *jockey* elements. The results showed a stable distribution of bands for all nine TEs among individual flies of the sublines (Fig. 2), as expected for an isogenic strain. Moreover, Bari and Seattle show the same pattern of I element fragments, while a clear heterogeneity is apparent among individual flies of Montpellier, consistent with results shown in Figure 1. Interestingly, the heterogeneity of the pattern of *I* fragments in Montpellier changed over time, as suggested by comparing Montpellier flies from different generations (M1, M2, M3). In addition to the *I* element, the hobo element showed a variable fragment pattern among individuals from the three sublines, in agreement with a recent study showing hobo instability in a *y*; *cn bw sp* line [24].

### FISH analysis of *I* elements distribution in isogenic lines

**Figure 3:**
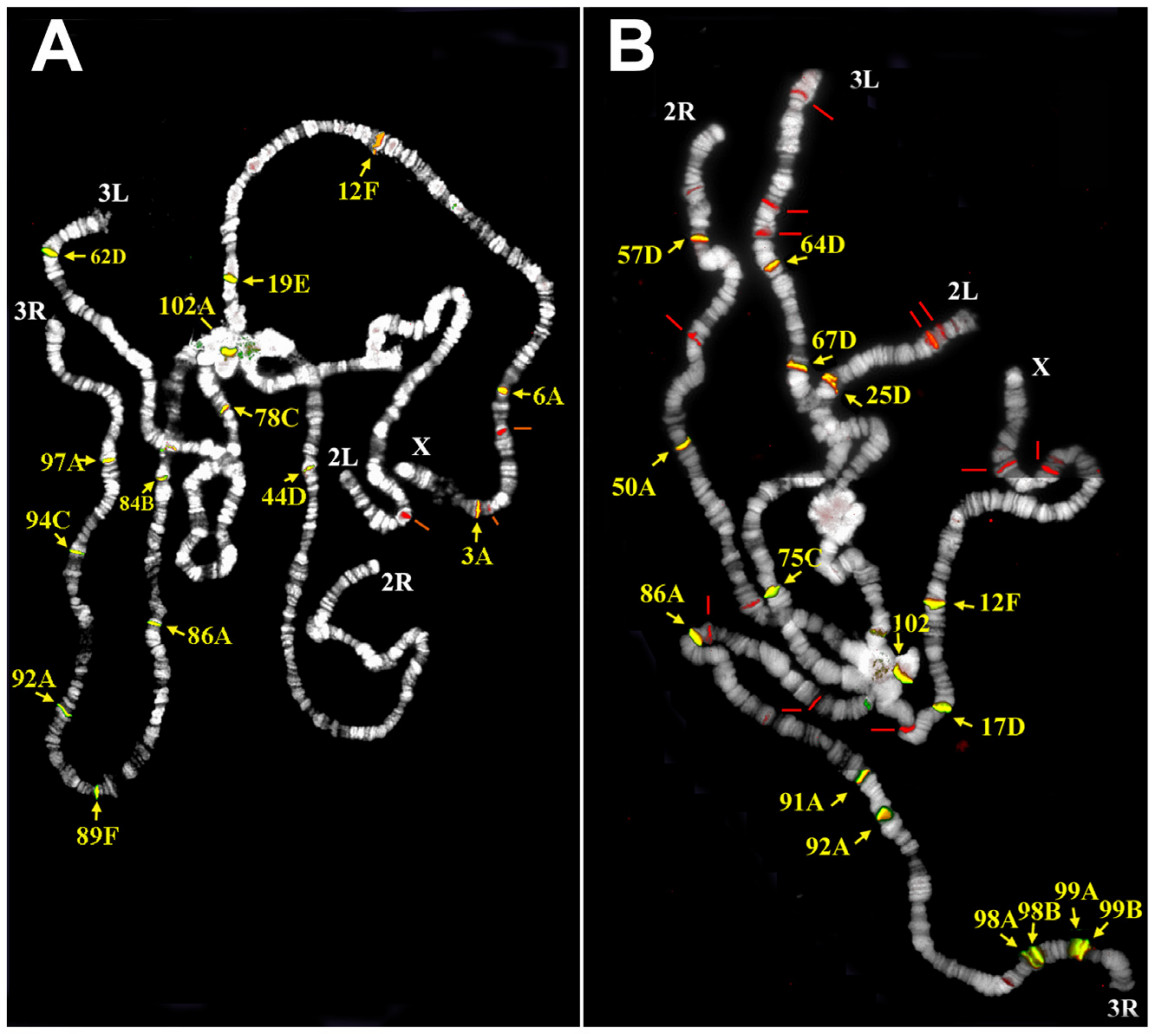
Fluorescence in situ hybridization analysis of *I* elements distribution in isogenic lines. Polytene chromosomes of Seattle and Montpellier strains were co-hybridized with *I*-3′ and *I*-5′ probes to compare the chromosomal distribution of *I* elements among the two sublines (see Material and Methods for details).

To further analyze the genomic distribution of the *I* elements in the *y; cn bw sp* sublines, we characterized at the cytological level the location of *I* copies using FISH on polytene chromosomes of Seattle and Montpellier sublines; the Bari subline was not tested given that the genomic distribution of *I* element-homologous fragments turned out to be identical to that found in Seattle in Southern assays. The results of the FISH analysis are shown in Figure 3. Table 2 summarizes the FISH sites found in the two sublines compared to those detected *in silico*. FISH sites homologous to *I* element 5′-specific probe are shown in green, those homologous to the 3′ end probe in red and those recognized by both 5′ and 3′ end probes in yellow. The number of I element sites that hybridize with both 5′ and 3′ probes is 14 in Seattle and 16 in Montpellier, while about 10 complete *I* elements are present in the *Drosophila melanogaster* annotated genome sequence [26]. Since the number of sites that hybridize with both 5′ and 3′ probes fits well with that of active I copies in an inducer strain [6], it is conceivable that most of them identify complete, or almost complete, *I* elements.

**Table 2:** Comparison of *I* element FISH mapping.

Strain	X	2L	2R	3L	3R	4	Total	3′ only
Seattle	3A6A12F*19E		44D	62D78C*	84B86A*89F92A94C97A	102A*	14	3
Montp	12F*17D	25D	50A57D	64D67D75C	86A*91A92A98A98B*99A99B	102A*	16	11
FlyBase	11A12F*19E		43E	66D78C*	86A*98B2*98B8	102A*	10	

Asterisks mark commun sites.

As shown in Figure 3 and Table 2, the cytological distribution of *I*-homologous sites clearly differs between Seattle and Montpellier. In addition, *I* hybridization sites in Seattle and Montpellier differ from those provided by the annotation of the sequenced genome, with only four sites being shared by all three genomes. Such dramatic differences in the chromosomal distribution of the *I* elements among sublines of the isogenic the *y*; *cn bw sp* strain provides cytological confirmation of the *I* element instability detected by Southern blotting. Retrotransposition of *I* elements is known to produce 5′ end truncated copies. Here, we find a higher number of signals seen only with the 3′ end probe (red) in Montpellier compared to Seattle, suggesting that the instability is associated with an increase in 5′ end truncated copies of I and thus with *de novo* retrotransposition.

### RT-PCR analysis of *I* element transcription in isogenic lines

**Figure 4:**
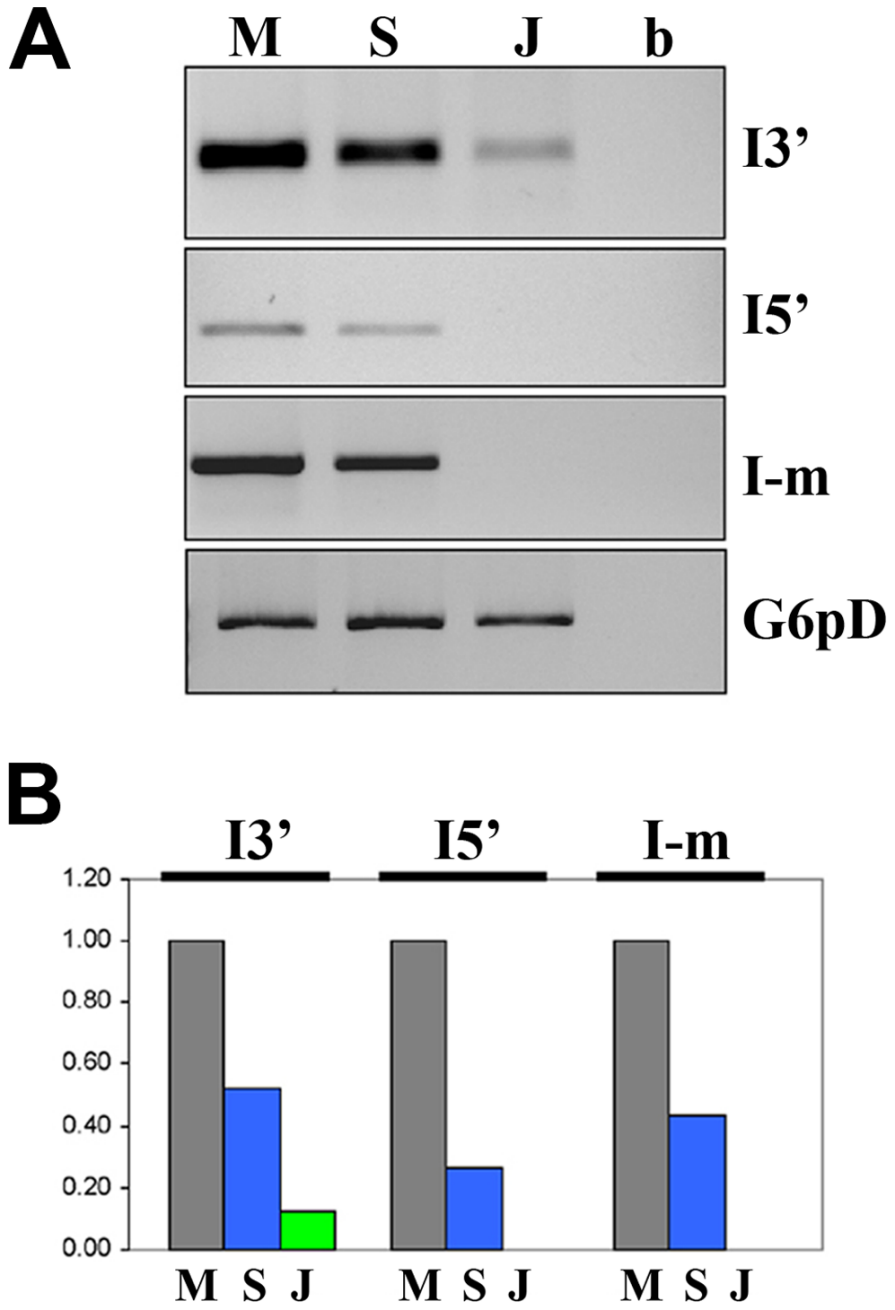
RT-PCR analysis of *I* elements. (A) Ethidium Bromide gel analysis of PCR products from reverse transcription of different portion of *I* element. *I*-3′ is a 351 bp fragment from position 4761–5112; *I*-m is a 574 bp fragment from position 2837–3411; *I*-5′ is a 699 bp fragment from position 22–721. Reference I element has Accession Number M14954. Adult females tested: Montpellier (M), Seattle (S), JA (J). G6pD is the internal control used for normalization. b is the blank of the PCR reaction. (B) Diagram of the quantitation of *I* transcripts showing the relative abundance with respect to the Montpellier value  = 1.

It was important to distinguish possible differences in the amount of *I* element transcripts between the Montpellier and Seattle sublines. These sublines were therefore analyzed by semi-quantitative RT-PCR, using for control the JA reactive (R) strain, which contains only defective, retrotranscriptionally inactive *I* elements located in pericentric heterochromatin. RT-PCR experiments were performed on totalRNA extracted from Montpellier, Seattle and JA adult females (Fig. 4). Using suitable primers (see Material and Methods), three different RT-PCR products were amplified corresponding to different portions of the I element: 5′ end (*I*-5′), 3′ end (*I*-3′) and an internal portion (*I*-m). *I*-5′ is a 699 bp fragment from position 22–721, *I*-m is a 574 bp fragment from position 2837–3411 and *I*-3′ is a 351 bp fragment from position 4761–5112. The results clearly show an increase of *I* element transcripts in adult females from Montpellier compared to both Seattle and JA. Such differences are seen for all *I*-5′, *I*-3′ and *I*-m fragments. In particular, compared to Montpellier Seattle contains about 30% of *I*-5′ transcripts and 50% of I-3′ transcripts. No *I*-5′ or *I*-m transcripts were found in the JA strain, as expected from a reactive strain mainly containing defective copies that have lost most of their 5′ and internal portion. The *I*-3′ transcripts seen in JA should reflect transcription of heterochromatin defective copies that maintain their 3′ end [2], [6].

**Figure 5:**
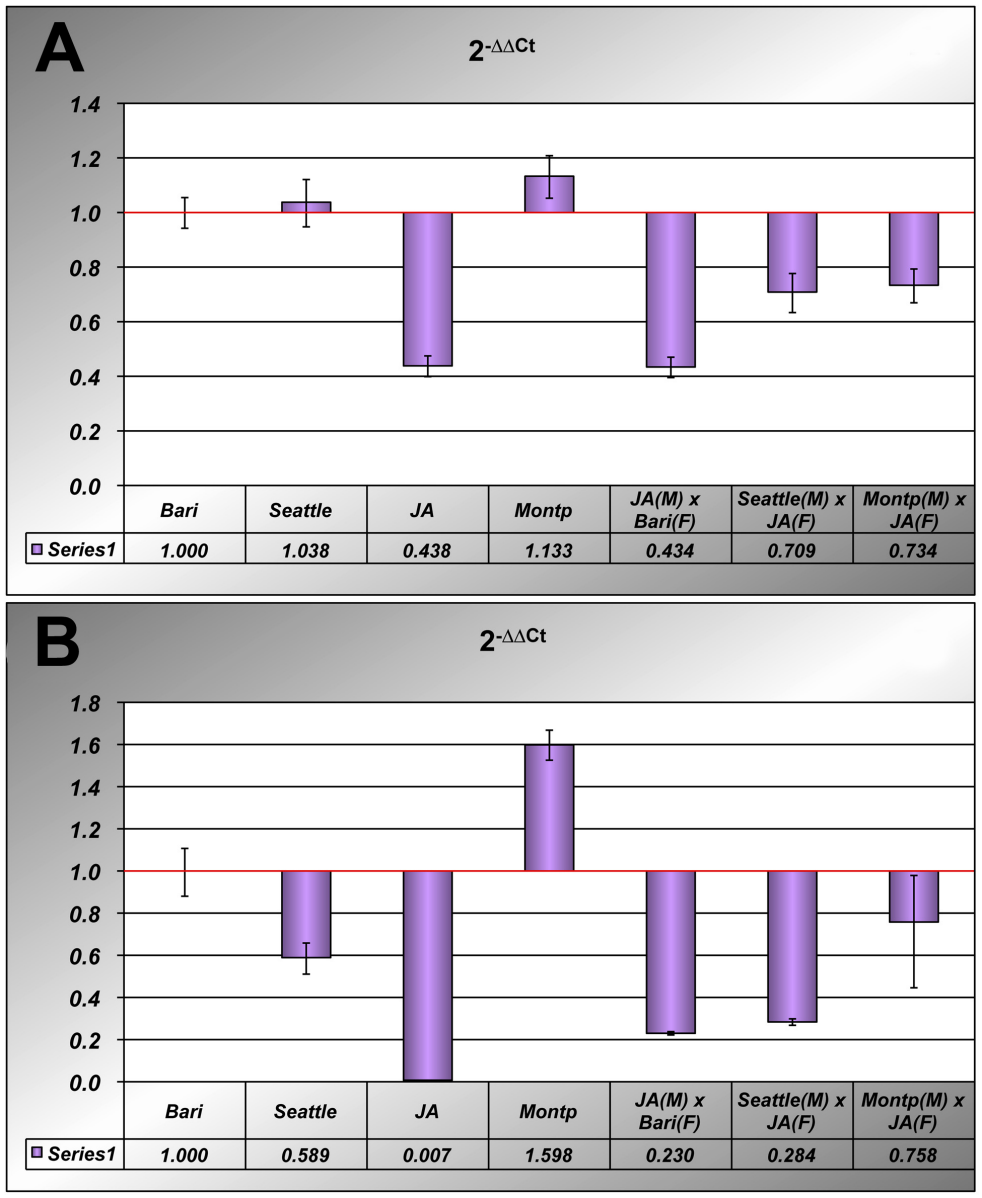
Real-Time PCR analysis of *I*-elements in gonads of three inducer stocks (Bari, Seattle, Montpellier), one reactive stock (JA), and from a non-dysgenic cross (JA male x Bari female) and two dysgenic crosses (Seattle male x JA female; Montpellier male x JA female. Transcripts calibration was performed as described in Mat and Meth using the 2^DDCt^ method and the Bari gonads as reference value. The relative amounts of *I*-5′ and *I*-3′ transcripts are shown in (A) and (B) respectively.

We sought to ascertain the differences detected thus far in the amount of *I* element transcription in real time RT-PCR assays of increased sensitivity. In addition to *y*; *cn bw*; *sp* sublines and JA (R) strain, we also analysed RNA from dysgenic and non dysgenic female gonads. Real time RT-PCR experiments confirmed and extended the quantitative PCR results (Fig. 5A): the highest amount of *I*-5′ end derived transcripts was found in Montpellier female gonads, while the lowest amount was found in gonads from JA females and from non-dysgenic females born from the crosses between JA males and Bari females. Notably, the amount of *I*-5′ transcripts increases significantly in dysgenic females gonads compared to both the JA and the non-dysgenic gonads, as one would expect, yet does not reach the level found in Montpellier. The highest amount of *I*-3′ transcripts was also found in Montpellier (Fig. 5B) and was significantly different from that exhibited by Bari and Seattle sublines. The gonads of JA females lack *I*-3′ transcripts almost completely, while those of non-dysgenic females from the crosses between JA males to Bari females, show low levels of such transcripts. Again, as for *I*-5′ transcripts, *I*-3′ transcripts increase in dysgenic crosses, though not reaching the Montpellier level. Notably, the gonads of dysgenic females born from the cross between JA females and Montpellier males showed a 2.5-fold increase in *I*-3′ derived transcripts compared to those born from cross between JA females and Seattle males.

## Discussion

In the present work, we used a combination of molecular and cytological approaches to study the activity of *I* elements in 3 sublines of the inducer *y*; *cn bw*; *sp* isogenic strain and in dysgenic and non-dysgenic backgrounds.

Earlier studies suggested that *I* elements and other TEs can be unstable in natural populations and in isogenic lines of *Drosophila melanogaster* [19], [20], [21], [22], [23]. Overall, the results of our cytological and molecular analyses clearly show that I elements are highly unstable in the Montpellier subline in the absence of classical dysgenic conditions. Moreover, our findings confirm and extend the cytological observations by Zhakarenko et al [24], who detected instability of TE families in the *y*; *cn bw*; *sp* isogenic strain. In particular, we now report that, at all levels of analysis, the genomic distribution of *I* elements differs among sublines of the isogenic *y*; *cn bw sp* strain. Compared to Bari and Seattle sublines, which exhibit the same I element distribution, Montpellier clearly showed instability of *I* elements. Such instability appears to be correlated to the amount of 5′ and 3′ *I* element transcripts detected by quantitative and real time RT-PCR.

Maternally transmitted I-element piRNAs play a pivotal role in controlling the stability of potentially active I element copies [17], [18]. Thus, it is possible that mutations affecting, directly or indirectly, factors controlling piRNAs formation segregate in the *y*; *cn bw*; *sp* inducer strain and eventually give rise to instability of *I* element and other TEs. For example, it has recently been found that Hsp90 mutations affect the biogenesis of *I* element piRNAs in *Drosophila melanogaster* and result in *I* element instability [27]. Alternatively, the molecular basis responsible for the I element instability observed here might differ somehow from that underlying *I* element transposition in dysgenic crosses. In other words, maternally transmitted *I*-element piRNAs may not be the only genetic determinants in control of *I* element mobility in *Drosophila melanogaster* strains. Dysgenic females of the I-R system are characterized by a decreased fertility related to the levels of reactivity and depending on the effects of *I* element retrotransposition in female germline [2], [6]. It is intringuing that the Montpellier subline shows no significant reduction of fertility, as one would expect from the high instability and high levels of transcription of *I* elements. However, we have observed that Montpellier sublines are frequently lost (data not shown), and it is tempting to speculate that this may result from a burst of *I* element retrotransposition that may affect fertility and viability.

Whichever molecular mechanism underlies the instability of *I* elements detected in the Montpellier subline, the observation that *I* element transcripts are more abundant in the gonads of Montpellier females compared to those of dysgenic females seems somehow paradoxical. It can be argued that the RNA products detected by RT-PCR may not only comprise functional *I* element-specific RNA intermediates, but also heterogeneous RNA species that would not affect the rate of retrotransposition. *I* element transcripts have indeed been detected by FISH in ovaries of *I* strains [17], but they are degraded and do not localize in the egg like the functional transcripts found in dysgenic females. Additional experiments will be required to investigate in more depth the molecular nature of I element instability in Montpellier sublines.

Genomes of different organisms can repress mobility of TEs using different control mechanisms. Such controls, however, can be bypassed by TEs, as shown here for the Montpellier subline. These interactions are part of a long-run “evolutionary game” of selection and adaptation, during which genomes can evolve new regulatory controls that may generate in turn new adaptative responses from TEs and viceversa.

## Materials and Methods

### Drosophila Strains

The name of Bari (B), Montpellier (M) and Seattle (S) sublines represent the locations of the labs where *y*; *cn bw*; *sp* flies where shipped directly from the Bloomington stock center and maintained for years.

### Southern analysis

DNA extraction, gel electrophoresis, transfer and hybridization were performed as previously described by Junakovic [28].

### Quantitative reverse transcription analysis

Total RNA was extracted from 40 adult females, 5 days old after eclosion, or from 50 gonads with the High Pure RNA Tissue Kit (Roche). Absence of contaminating DNA was always checked by PCR. 1 µg RNA was reverse transcribed with SuperScript II (Invitrogen) using oligo(dT)15 as primer and in accord to the protocol of the manufacture. Quantitative PCR shown in Figure 4 was performed with 1 µl cDNA in a 50 µl Platinum Taq Polymerase mix (Invitrogen) and in an exponential amplification condition (28 cycles) as determined in pilot experiments. 15 µl were loaded on a 1,5% agarose gel, and the areas of ethidium bromide bands were determined by scanner densitometry. The three couple of *I* element primers were as follows: *I*-3′, I_4761U 5′ CCAAACATAAATACCACAGA and I_5112L 5′ AGTTTTTGTATGTTATCTGGA; *I*-5′: I_22U 5′ AGAGATAAGTCGTGCCTCTC and I_721L 5′ GTACTCGGACTGTTTCGTAC; *I*-m (middle) I_2837U 5′ GTATCTAGAACTTAGCTCAGCAC and I_3411L 5′ GACTAGTGGCTTGATGTATGCGG. Primers for the G6pD used as internal control were G6pD_U 5′ AGTCGCCTACAATGGTCTGC and G6pD_L 5′ GTTCGAATCGTTGCTAACGG. Quantitative Real Time RT-PCR (Figure 5) was achieved with 1 µl cDNA obtained from RNA gonads as described above and analysed with the 7300 Real Time PCR System (Applied Biosystems) in a total volume of 25 µl using SYBR Green PCR Master Mix (Applied Biosystems). The relative expressions of *I* were obtained with the following couple of primers: *I*-5′, 5′-TTTGCCTGTGGAGGAGAAGT and 5′-TTAGCAGGTTGCCGTCTCTT (position 331–388); *I*-3′: 5′-GACCTTGCGACAAAACAGAA and 5′-GCATGGGTGTGAGGTGTTC (position 4803–4886). The coordinate of PCR primers are from the complete I element present in the sequence with AC M14954. Values were normalized with the expression of G6PD internal control amplified with the following primers: G6pD_f: 5′ ACCGCCCTGGATCTCATAAT and G6pD_r: 5′ CAAAGATGACGAACGTGTGC. For quantitation of the transcripts, the 2DDCT method was used [29].

### Probes

Sequences internal to TEs have been described previously [28]. The two clones pI770 and pI771 containing the 5′ and the 3′ portions of the *I* element [1] were used as probes.

### In Situ Hybridization

Probes were labeled by nick-translation with Cy3-dCTP or FluorX-dCTP (GE Healthcare). Polytene chromosomes prepared as described by Pardue [30] were stained with DAPI, 4′, 6′-diamidine-2′-phenylindole-dihydrochloride. Digital images were obtained using an Olympus epifluorescence microscope equipped with a cooled CCD camera. Gray scale images, obtained separately recording Cy3, FluorX and DAPI fluorescence by specific filters, were pseudo colored and merged for the final image using the Adobe Photoshop software.
